# Education Research: A Qualitative Study of Transformative Experiences in Neurology Residency

**DOI:** 10.1212/NE9.0000000000200253

**Published:** 2025-09-30

**Authors:** Ema V. Karakoleva, Annika Daya, Max R. Lowden

**Affiliations:** 1 Pennsylvania State University College of Medicine, Hershey, PA;; 2 Beth Israel Deaconess Medical Center, Harvard Medical School, Boston, MA; and; 3 Department of Neurology, Pennsylvania State University-Milton S. Hershey Medical Center, Hershey, PA.

## Abstract

**Background and Objectives:**

Professional identity formation during residency is often catalyzed by emotionally complex experiences that extend beyond structured curricula. This study explores how neurology residents navigate autonomy, mentorship, and workload during training, using transformative learning theory to examine identity formation beyond skill acquisition.

**Methods:**

We conducted a qualitative study at a US academic neurology residency program. Participants included current PGY-3 and PGY-4 residence and recent alumni (graduated 208–2021) from a single four-year ACGME-accredited neurology program. Individuals were recruited through email and completed demographic surveys before participating in a 45-minute Zoom interview. Interviews were guided by a semistructured protocol and explored expectations, stressors, mentorship, and pivotal learning moments. Transcripts were analyzed thematically using MAXQDA. Two independent, blinded coders applied a shared codebook and achieved interrater reliability (Cohen κ ≥ 0.70). Reflexive journaling and interdisciplinary team debriefs enhanced analytic rigor.

**Results:**

Twenty-one participants (mean age 35.4 ± 5.3 years; 57% female; 48% White; 38% DO, 43% MD) completed interviews. Three intersecting domains of transformation emerged (1) balancing autonomy and mentorship, (2) adapting to high workload, and (3) discovering meaning in clinical work. Most participants described their development as nonlinear, driven by “selective emulation” of mentors and emotionally charged scenarios requiring reflection and adaptation. The majority (67%) reported growth through a blend of autonomy and mentorship. Coping strategies for workload were predominantly self-reliant (62%) or relational (57%), with fewer citing institutional supports (33%). Meaning was most often derived from patient-centered care (67%), followed by alignment with a subspecialty (43%) or systemic contributions through teaching or advocacy (33%).

**Discussion:**

This study provides a theoretically grounded framework for understanding identity formation in neurology residency. High-responsibility clinical moments were reframed as developmental turning points when paired with reflection and supportive mentorship. Findings underscore the value of psychologically attuned environments that facilitate adaptive coping, meaning-making, and progressive autonomy. Limitations include single-site sampling and potential recall bias among alumni. Future research should explore longitudinal identity trajectories and examine how early meaning-making predicts later professional fulfillment and resilience.

## Introduction

Residency is intended to provide structured preparation for independent clinical practice, emphasizing both competence and professional development. However, variability across programs in clinical exposure, mentorship, and supervisory style has led to differences in how residents experience training.^1-4^ In neurology specifically, such variation is particularly salient due to the specialty's diagnostic complexity, emotionally charged scenarios, and longitudinal patient care.^5-7^ Although standardized milestones and competencies provide a common framework, implementation differs widely, and the impact of this variability on resident development remains underexplored.

Professional identity formation and the development of clinical autonomy are central to the transition from trainee to independent practitioner.^4^ Together, they shape how residents approach uncertainty, internalize responsibility, and develop confidence in decision-making. Without adequate support, these processes can be hindered, affecting not only learning outcomes, but also resident well-being and patient care.^4,8,9^ Although previous studies have examined burnout, satisfaction, and perceived preparedness,^6,10^ fewer have focused on the deeper, formative learning experiences through which identity and autonomy evolve.

In addition to program structure, residents' growth is influenced by factors such as mentorship quality, feedback consistency, and the broader learning environment.^11-14^ Barriers such as inconsistent evaluation, limited formative feedback, and cultural hierarchies can constrain development, particularly for residents navigating stress, transition, and personal uncertainty.^10,15^ Furthermore, personal life experiences, including educational debt, career decision-making, and resilience in the face of burnout, significantly shape how residents engage with training.^6,7,16^ Together, these educational and personal dimensions contribute to how trainees construct meaning, adapt, and prepare for independent practice.

Transformative learning theory, first introduced by Mezirow,^17^ posits that adult learning occurs through disorienting dilemmas that prompt critical reflection and restructuring of internal meaning frameworks. This theory has been applied to understand how emotionally salient clinical experiences can catalyze personal and professional growth.^18,19^ In medical education, such transformative moments can foster development of traits such as adaptability, reflective capacity, and a more integrated professional identity, which are particularly poignant in neurology. Applying this lens offers a way to explore how residents interpret and integrate experiences that shape their transition to autonomy.

This study explores the transformative experiences of neurology residents during their early years of training to understand how identity, autonomy, and perceived readiness for practice are constructed. Because transformative experiences are often nuanced, emotionally salient, and highly individualized, we used a qualitative approach to capture the depth and complexity of residents' reflections.^20^ Through qualitative analysis of resident reflections, we aim to identify the emotionally meaningful and professionally formative experiences that contribute to growth. By characterizing these developmental processes, we hope to inform educational strategies that support not only competence but also long-term adaptability and resilience in early career neurologists.

## Methods

### Standard Protocol Approvals, Registrations, and Patient Consents

The study protocol was reviewed by the Penn State College of Medicine Institutional Review Board (IRB) and determined to be exempt from full review because it did not involve identifiable personal health information (IRB Protocol #00019853). All participants provided informed consent electronically through REDCap before data collection.^21,22^ The study adhered to the Consolidated Criteria for Reporting Qualitative Research (COREQ) guidelines (eTable 1).

### Study Design

This qualitative, explorative study aimed to explore the residency experiences of neurology trainees. Data collection was conducted at the Penn State College of Medicine (PSCOM) through semistructured, one-to-one interviews conducted through Zoom. The study used thematic analysis, with themes derived inductively from the data.

### Research Team and Reflexivity

Interviews were conducted by 2 female medical students (E.V.K. and A.D.) who were in their second and third years of training, respectively, at the time of data collection. Neither interviewer was affiliated with the neurology residency program, and both were unknown to participants before recruitment. This helped minimize bias and preexisting relationship influence. Participants were aware that the researchers were medical students conducting a research study on residency experiences and that they held no evaluative role in their program. Both interviewers received formal training in qualitative interviewing in collaboration with the Qualitative and Mixed Methods Core (QMMC) at PSCOM. Training included shared use of a semistructured interview guide (eAppendix 1), mock interviews, and calibration meetings to ensure consistency and fidelity to the study objectives. The interviewers shared an interest in medical education and professional development, which shaped their engagement in the research.

### Participant Selection and Recruitment

Participants were selected from a list of current neurology residents, fellows, and alumni provided by the neurology residency coordinator at Penn State Health Medical Center (PSHMC). A purposive sampling strategy was used to recruit postgraduate year-3 (PGY-3) and PGY-4 neurology residents, as well as physicians who had completed residency or fellowship at PSHMC between 2018 and 2021. PGY-1 and PGY-2 residents were excluded because PGY-1 residents were primarily completing internal medicine training and PGY-2 residents were still earl in their neurology adjustment phase. The selection focused on participants who had sufficient exposure to neurology training and the opportunity to reflect on professional development. Although both PGY-3 and PGY-4 residents were eligible, all resident participants interviewed were in their PGY-4 year at the time of data collection.

Potential participants received an email invitation containing a REDCap link, where they could review the IRB-approved Summary Explanation of Research and provide electronic consent (eConsent). After consent, they completed a demographic questionnaire and shared contact information. A study team member followed up within 24 hours to schedule a Zoom interview. A total of 21 participants were interviewed; one additional individual consented but did not respond to scheduling efforts.

### Interview Process

Interviews were conducted through Zoom between July 2022 and June 2023. Each interview lasted approximately 45 minutes and involved only 1 participant and 1 interviewer. Interviews followed a semistructured guide developed in collaboration with QMMC and pilot tested internally. The guide included open-ended questions exploring residency expectations, personal and professional challenges, transformational experiences, support systems, and future goals (see eAppendix 1).

All interviews were audio-recorded with participant permission and professionally transcribed. Transcripts were deidentified and uploaded to REDCap for secure storage, with all identifying details removed. Field notes were not systematically collected as part of the study protocol. No repeat interviews were conducted. Thematic consistency was observed across the final interviews, suggesting that data saturation was likely achieved. To acknowledge their participation, each participant was offered a $10 ClinCard sent through mail.

### Data Analysis

Interview transcripts were analyzed using MAXQDA software (VERBI Software, 2022),^23^ and demographic data were managed in Excel (v2407, Microsoft, Redmond, WA). A codebook was developed inductively through team discussion. Two independent coders applied the codebook using NVivo 12.0. Thematic codes were derived from the data rather than predefined. Interrater reliability was ensured using Cohen kappa (≥0.70).

The coding tree was refined iteratively as themes emerged. The final overarching themes were derived inductively from the coded data through repeated team discussions and consensus. Although the team had a general awareness of prior literature on resident development, these themes were not predefined and reflected patterns that consistently appeared across participant narratives. Direct quotations were selected to illustrate key findings and were attributed using anonymized participant numbers. Participants did not review transcripts or comment on final themes.

### Data Availability

Anonymized data not published within this article will be made available by request from any qualified investigator.

## Results

### Demographic and Educational Background of Survey Participants

A total of 21 participants completed the survey. Participant characteristics are summarized in eTable 2. The mean age was 35.4 ± 5.3 years (range 28–45), with 12 (57.1%) identifying as female and 10 (47.6%) as White. Nearly all participants (n = 18, 86%) completed their neurology residency training at Penn State Health Milton S. Hershey Medical Center, a four-year categorical neurology residency program accredited by the ACGME. Participants held the following degrees: Doctor of Medicine (MD; n = 9, 42.9%), Doctor of Osteopathic Medicine (DO; n = 8, 38.1%), MD as International Medical Graduates (IMGs; n = 3, 14.3%), and MD with a Doctor of Philosophy (MD/PhD; n = 1, 4.8%). At the time of interview, most were attending physicians (n = 14, 66.7%), while others were fellows (n = 4, 19.0%) or current residents (PGY-4; n = 3, 14.3%). The distribution of participants by age and sex is illustrated in eFigure 1, with the 36–40 age group comprising the largest proportion (33.3%).

### Transformational Themes in Early Residency

**Figure 1:**
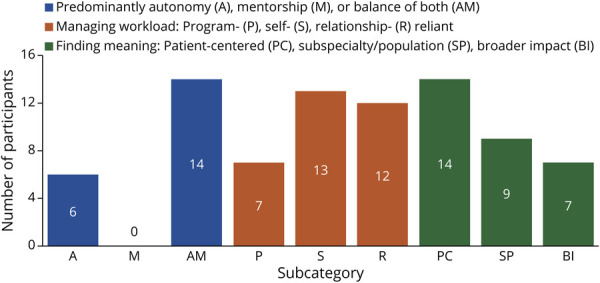
Distribution of Participants Across Subcategories Within 3 Major Themes of Transformation Bar plot showing the number of participants (N = 21) endorsing each subcategory within the 3 thematic domains: autonomy and mentorship (A, M, AM), workload adaptation (P, S, R), and finding meaning in clinical work (PC, SP, BI). Subcategories include autonomy (A), mentorship (M), autonomy and mentorship (AM), program-oriented strategies (P), self-reliant strategies (S), relational strategies (R), patient-centered care (PC), subspecialty or population alignment (SP), and broader impact (BI).

Qualitative analysis revealed 3 primary themes of transformation during early neurology residency: (1) balancing autonomy and mentorship, (2) adapting to workload demands, and (3) discovering meaning in clinical work. These themes reflect distinct yet interconnected areas of growth as residents progressed from junior to more senior roles. Each theme includes specific subcategories, resulting in 9 total subthemes. Major themes and subcategories are summarized in Table 1. Definitions, representative quotes, and frequencies of themes are summarized in Table 2. The distribution of participants across each of these 9 subcategories is depicted in Figure 1. Below, we elaborate on each theme derived from participant narratives.

**Table 1:** Transformative Learning Themes and Subcategories

Transformative learning theme	Subcategories
Balancing autonomy and mentorship	Autonomy (A), mentorship (M), autonomy, and mentorship (AM)
Adapting to workload demands	Program-oriented (P), self-reliant (S), relational (R)
Discovering meaning in clinical work	Patient-centered (PC), subspecialty-driven (SP), broader impact (BI)

**Table 2:** Key Transformative Themes, Subcategories, Descriptions, and Representative Quotes From Participants

Theme	Subcategory	Code	Description	Participant quotes	Frequency of identified Subcategory, n (%)
Balancing Autonomy and Mentorship	Autonomy	A	Preferred self-directed learning and independence	“The night float rotation at the VA makes you think on your feet… makes you realize that you actually know a lot of what you're doing.”	6 (29%)
	Mentorship	M	Emphasized support and guidance from mentors	“They [the senior team members] were always available when I needed them.”	0 (0%)
	Autonomy and mentorship	AM	Found transformation through a balance of independence and support	“You're by yourself in the hospital… no matter how prepared, you still have anxiety. But the attendings were really friendly and reachable.”	14 (67%)
Adapting to High Workload	Program-oriented	P	Managed workload through systemic or institutional strategies	“Culture has changed… the Senior Residents started coming in every weekend. That made a huge difference.”	7 (33%)
	Self-reliant	S	Developed personal strategies to adapt to stress	“I used to talk so fast… that may be the reason people didn't listen. So I worked on my communication skills.”	13 (62%)
	Relational	R	Leaned heavily on peers and social support networks	“My co-residents… they kept me afloat.”	12 (57%)
Finding Meaning in Clinical Work	Patient-centered	PC	Fulfillment through individual patient care relationships	“Knowing… that you helped someone… is extremely fulfilling. Be a human helping humans. Listen to your patient.”	14 (67%)
	Subspecialty/population-driven	SP	Found meaning in specific diseases or patient populations	“I'm not only here to learn neurology. I'm here to grow into the kind of doctor I would want for my own family.”	9 (43%)
	Broader impact	BI	Motivated by teaching, advocacy, systems reform, or leadership	“You want to help make things better for the next generation of residents.”	7 (33%)

Abbreviations: A = autonomy; AM = autonomy and mentorship; BI = broader impact through teaching, advocacy, or leadership; M = mentorship; P = program-oriented strategies; PC = patient-centered meaning; R = relational strategies; S = self-reliant strategies; SP = subspecialty/population-driven meaning.

This table outlines the 3 primary themes identified through thematic analysis, along with their associated subcategories. Each subcategory is accompanied by a brief description, a representative participant quote, and the number (and percentage) of participants who referenced the subtheme during interviews.

#### Balancing Autonomy and Mentorship

Most residents (n = 14, 67%) described their development as shaped by a combination of autonomy and mentorship (Group AM), while others (n = 6, 29%) emphasized autonomy alone (Group A). No participants identified mentorship in isolation (Group M) as their primary driver of growth.

Autonomy-driven learning was defined as growth through independent responsibility and decision-making, often in high-responsibility or unsupervised settings such as night float. Residents described gaining confidence by managing complex situations on their own:

You learn the most when you’re out there by yourself, but knowing someone will pick up the pieces if needed—that’s how I grew.

There were situations where you had to figure it out on your own, and that was really uncomfortable… but also the times I remember learning the most.

Mentorship referred to structured support that offered psychological safety, scaffolding, and feedback, especially during early transitions. Residents noted that effective mentorship created space to take risks while feeling supported:

I liked that my attendings gave me the space to make decisions but were always there to back me up. That balance made me feel safe but also pushed.

One of my mentors let me run the list on my own pretty early. It was terrifying, but that trust helped me realize I was capable.

Blended Autonomy and Mentorship development (Group AM) captured the integration of both autonomy and mentorship. Residents often described learning by observing multiple attendings and selectively adopting behaviors that shaped their own clinical style and identity:

I watched how different attendings handled tough conversations, and I tried to take the best parts from each one. That’s how I developed my own style.

The mentorship wasn’t just about medicine—it was about how to be a person in this field, how to carry yourself.

#### Adapting to High Workloads

Residents described a range of strategies for managing the demands of residency, categorized into 3 subthemes: self-reliant (Group S, n = 13, 62%), relational (Group R, n = 12, 57%), and program-oriented or structural strategies (Group P, n = 7, 33%).

Self-reliant strategies involved personal coping mechanisms developed independently, including emotional regulation, time management, and task prioritization:

There are some tough rotations where the workload feels overwhelming, but I tried to stay focused on what I could control and just keep going. I learned a lot about myself during those months.

I think I got stronger because I didn’t back away from the hard stuff. It was hard, but I kept my head down and got through it.

Relational strategies referred to support drawn from interpersonal relationships, including coresidents, mentors, family, and friends, which helped buffer stress, with many residents emphasizing the emotional grounding they gained from shared experience:

What helped most was knowing we were all in this together.

There’s something grounding about knowing you’re not the only one struggling. Just having someone to vent to in the call room made a big difference.

Structural strategies referred to program-level or system-based changes that helped mitigate workload, such as improved onboarding or adjusted schedules:

I think a better onboarding process made a big difference. I felt like I had a roadmap, which lowered the stress.

The program made some changes like adjusting the call schedule, and honestly, it helped. It’s small, but it gave me space to breathe.

#### Finding Meaning in Clinical Work

Nearly all participants (n = 20, 95%) identified meaningful aspects of their work that helped sustain them through training. These clustered into 3 subthemes: patient-centered care (Group PC, n = 14, 67%), subspecialty or population alignment (Group SP, n = 9, 43%), and broader systemic impact (Group BI, n = 7, 33%).

Patient-centered care, the most frequently cited source of meaning, referred to meaning derived from continuity, emotionally resonant encounters, and individual relationships with patients:

You're seeing the decisions you made and how they impact a patient… it's transformational.

You treat patients, and you see them get better in front of your eyes… that’s what I love about stroke.

Subspecialty or population alignment emerged when rotations resonated intellectually or emotionally, helping residents clarify long-term goals:

During my epilepsy rotation, I felt like I found my people. The cases were complex, and I enjoyed the detective work—it helped solidify my career direction.

Working with the stroke team made me feel aligned with a mission—it wasn’t just clinical care; it was about acting quickly and making a difference in real time.

Broader systemic impact described fulfillment derived from activities beyond bedside care, particularly teaching, advocacy, and leadership. These roles fostered agency and a sustained sense of purpose:

I’m more and more passionate about teaching [neurology] because I love it so much. I get to teach medical school students now, and also residents where I work, and I really love it.

I think that residents should be supported a little bit more… I don't think that we're adequately represented in a lot of decisions that are made… when we are on the frontline.

## Discussion

This study provides a nuanced account of the transformative processes that shape early neurology residency, demonstrating how clinical identity is forged not simply through knowledge acquisition, but through the dynamic interplay of autonomy, mentorship, emotional adaptation, and meaning-making. By organizing residents' reflections across 3 intersecting axes: (1) autonomy and mentorship, (2) coping with workload, and (3) sources of meaning, this work offers a multidimensional framework for understanding personal and professional development during one of the most formative stages of medical training. Our findings extend current conversations in medical education by highlighting how residents actively construct their identities through selective emulation, emotional processing, and meaning-making, rather than passively absorbing professional norms.

This study contributes to emerging scholarly conversations about how competency-based medical education (CBME) might be enriched by greater attention to residents' identity development and emotional experiences. Although CBME provides essential structure and shared expectations for performance, our findings suggest that the process of becoming a physician also involves navigating dimensions of professional development that may fall outside formal milestones, such as ambiguity, emotional dissonance, and meaning-making.^4,24,25^ Rather than viewing experiential learning and CBME as competing paradigms, we propose they are complementary: a better understanding of how residents interpret and integrate clinical experiences can inform the environments and mentorship practices that enable CBME goals to be met more holistically.^26,27^

Although CBME has provided essential structure and shared expectations for residency training, some aspects of professional growth, such as emotional adaptation, identity formation, and reflective learning, may receive less emphasis in current implementations.^4,8,24,25^ Our study participants described transformation as arising not just from technical skill-building, but from emotionally charged experiences, reflection, and mentorship. We offer transformative learning theory as a complementary lens to illustrate how these disorienting moments can catalyze growth. Although this theory has been explored in undergraduate medical education and international contexts,^19,20,28^ it remains underutilized in US residency training, particularly in neurology. Supporting these formative experiences may help CBME more fully develop reflective and resilient physicians.

Participants frequently described their development as a gradual transition from dependency to independence, facilitated not by a sudden removal of scaffolding, but by mentorship that adjusted responsively to their evolving confidence. Our findings complicate traditional binaries between supervision and autonomy, echoing recent critiques of the Entrustable Professional Activities (EPA) model for oversimplifying the learning process.^26^ Rather than progressing linearly, residents described an iterative process of trial, reflection, feedback, and adaptation.

Moreover, our participants engaged in what we term *selective emulation*: constructing a professional identity by integrating favored traits from various mentors. This process supports sociocultural theories of identity formation, which view learning as embedded in communities of practice and mediated by observation, interaction, and meaning-making.^4,27^ These dynamics are often absent from formal curricular evaluations but are central to how residents navigate hidden curricula and resolve identity tensions.

Programs might consider adopting mentorship structures that support this flexible, relational learning. Layered mentorship, including incorporating peer, near-peer, and faculty guidance, can create psychologically safe environments in which learners can explore, adapt, and self-author. Faculty development should also address the emotional dimensions of mentorship, encouraging supervisors to remain attuned not only to clinical performance, but also to each resident's evolving identity and emotional well-being.

Although the emotional burden of early residency is well documented,^9^ our findings suggest that intense clinical workload can also serve as a powerful driver of professional growth. Residents described high-acuity scenarios, such as night shifts, ICU coverage, or emotionally charged cases not only as stressful, but as pivotal moments that challenged their clinical confidence and emotional preparedness. These experiences often disrupted their existing assumptions and required rapid adaptation, which they later identified as turning points in their development. This process aligns with transformative learning theory, which describes how individuals evolve through confronting difficult situations, critically reflecting on them, and ultimately reshaping their perspectives and professional identities.

What differentiates our findings is the *range of coping strategies* residents employed: some leaned into self-regulation, others into collegial support, and a smaller group cited programmatic structures. It is important that these were not static styles; participants moved fluidly between strategies depending on context and personal capacity. This adaptability suggests that psychological flexibility, not any single coping strategy, is the more meaningful indicator of resilience.

This variability resonates with transformative learning theory, which posits that significant growth often follows “disorienting dilemmas” that prompt reflection and restructuring of perspective.^17^ Rather than being defined by stress alone, residents' developmental trajectories were shaped by their ability to process and learn from it. That few residents cited institutional solutions reinforces prior critiques that wellness infrastructures in GME are underdeveloped, and often reactive rather than preventive.^29,30^ Our findings suggest that programs must do more than monitor wellness; they must explicitly teach adaptive strategies, normalize help-seeking, and create reflective spaces to metabolize difficult clinical experiences into professional growth.

Programs must recognize that although high-acuity clinical exposure is essential for skill-building, it must be paired with intentional supports, including peer debriefing, reflective spaces, and wellness resources, to ensure that disorienting dilemmas catalyze growth, rather than disengagement.

One of the most striking patterns in our data was the central role of meaning-making in sustaining residents through burnout-prone phases. This aligns with prior literature on “career calling,” defined as a sense of deep purpose or meaningful direction in one's professional life, and intrinsic motivation,^31,32^ both of which have been associated with greater resilience and job satisfaction. These results affirm the importance of designing programs that help trainees connect their daily clinical work with personal values. It is important that residents in our study did not all derive meaning from a singular source. Some cited patient-centered care, others found inspiration in subspecialty alignment, and still others described broader roles in teaching, leadership, or advocacy as central to their sense of purpose.

What our study adds is detail about *how* meaning is constructed: through reflection, narrative reconstruction, and alignment with personal values. For many, emotionally taxing experiences, such as delivering bad news or navigating ethical uncertainty, were reframed as transformative. These were not merely tolerated; they became formative through the lens of purpose.

Residency programs can support this process by incorporating narrative medicine, providing career advising that explores value alignment, and creating space for residents to connect emotionally to their work beyond just cognitively.^9,33^

Our study contributes a taxonomy of developmental experiences grounded in transformative learning theory and expands its application to early residency. Notably, we found that growth was not unidirectional or static; rather, residents moved fluidly between coping styles, mentorship preferences, and sources of meaning. This flexibility suggests that adaptability, and not any one fixed trait, is a key outcome of early residency. Future work should explore how these transformations evolve longitudinally across training and whether early meaning-making predicts later professional satisfaction or burnout. In addition, investigating how programs can proactively tailor mentorship structures or match residents to preferred coping resources may help optimize both performance and well-being.

As researchers, we acknowledge that our positionalities shaped this inquiry. The lead authors conducted interviews as senior medical students with shared lived experience of clinical training, which may have facilitated openness but also introduced social desirability bias. Throughout analysis, we engaged in reflexive journaling and regular debriefs with the QMMC to bracket assumptions and enhance analytic rigor. Our team's interdisciplinary composition (medical students, neurology faculty, and qualitative research consultants) provided a valuable check on positional bias and enriched the interpretive process.

A major strength of this study is its theoretically grounded and methodologically rigorous design, which enabled in-depth exploration of underexamined aspects of professional identity formation in neurology residency. Credibility was enhanced through thematic saturation, dual coding, and reflexivity practices. Although all participants were affiliated with the same institution at the time of recruitment, they varied in their training pathways. Some completed only residency, others completed only fellowship, and several completed their intern year elsewhere. This introduced some heterogeneity in experience. Nevertheless, the single-institution setting remains a key limitation because local program culture and structure may have shaped participants' experiences in ways that constrain generalizability. Future multi-institutional studies are needed to determine whether the transformative processes identified here hold across diverse training environments. Although the sample was diverse in educational background, sex, and race or ethnicity, future research should also intentionally recruit participants from underrepresented groups to better understand how systemic inequities influence transformative learning. In addition, although the inclusion of recent residency alumni provided valuable retrospective insights, it may have introduced recall bias because participants may have reconstructed past experiences with the benefit or distortion of hindsight. Notably, this study did not explicitly explore how exposure to different patient care environments, such as urban vs rural settings or varying levels of patient insurance coverage, may shape professional identity. Similarly, participation in advocacy or policy-related efforts at the local or national level may also contribute to identity development but was not a focus of this analysis. Future studies may benefit from examining how these contextual and structural factors influence transformative learning during residency.

In conclusion, this study offers a structured framework for understanding how early residency fosters identity transformation through dynamic strategies of learning, coping, and meaning-making. By applying transformative learning theory, we demonstrate that professional identity is not acquired in a linear path but shaped contextually through dissonance, reflection, and dialogue.

Future research should explore whether early meaning-making predicts long-term career satisfaction and how tailored mentorship, and reflective spaces can support identity development across specialties. As medical education evolves to cultivate not only competence but also character, our findings offer a conceptual and practical roadmap for fostering environments that develop both skill and self-awareness.

## Glossary

CBME: competency-based medical education
PSHMC: Penn State Health Medical Center
