# Education Research: A Qualitative Study of Transformative Experiences in Neurology Residency

**DOI:** 10.1212/NE9.0000000000200295

**Published:** 2026-01-27

**Authors:** Ema V. Karakoleva, Annika Daya, Max R. Lowden

In the Research Article “Education Research: A Qualitative Study of Transformative Experiences in Neurology Residency” by Karakoleva et al.,^1^ the second sentence of the Methods in the Abstract should read, “Participants included current PGY-3 and PGY-4 residents and recent alumni (graduated 2018–2021) from a single 4-year ACGME-accredited neurology program.” The authors regret the error.
